# Inflammasome Genes' Polymorphisms in Egyptian Chronic Hepatitis C Patients: Influence on Vulnerability to Infection and Response to Treatment

**DOI:** 10.1155/2019/3273645

**Published:** 2019-01-09

**Authors:** Shady Z. K. Estfanous, Sahar A. Ali, Sameh M. Seif, Sameh H. A. Soror, Dalia H. A. Abdelaziz

**Affiliations:** ^1^Department of Biochemistry and Molecular Biology, Faculty of Pharmacy, Helwan University, Ain Helwan, Helwan, Cairo 11795, Egypt; ^2^National Tropical Medicine and Hepatology Research Institute, Cairo, Egypt; ^3^Department of Comparative Biology and Experimental Medicine, Faculty of Veterinary Medicine, University of Calgary, Calgary, Alberta, T2N4Z6, Canada

## Abstract

Chronic inflammation is a pivotal contributor to the liver damage mediated by hepatitis C virus (HCV). The NOD-like receptor, pyrin domain-containing 3 (NLRP3) inflammasome is activated by HCV in both hepatocytes and Kupffer cells. The aim of our study was to investigate the association of nine single-nucleotide polymorphisms in four inflammasome genes (NLRP3, CARD8, IL-1*β*, and IL-18) with the susceptibility to HCV infection and outcome of interferon treatment in 201 Egyptian chronic hepatitis C patients and 95 healthy controls. The genotyping was conducted using TaqMan predesigned SNP assay. In the comparative analysis, the CC genotype of the NLRP3 rs1539019 was found to be associated with the lower risk to chronic HCV infection (OR: 0.33, 95% CI: 0.17-0.62). This association was also found for the CA genotype and the A allele of the NLRP3 rs35829419 (OR: 0.18 and 0.22, respectively), in addition to the GG genotype and G allele of IL-18 rs1946518 (OR: 0.55 and 0.61, respectively). In contrast, the AA genotype of the IL-1*β* rs1143629 was significantly more frequent in HCV patients (OR: 1.7, 95% CI: 1-2.86). Notably, the frequency of the AA genotype of NLRP3 rs1539019 was significantly higher in patients with lack of response (NR) to the interferon treatment (OR: 1.95, 95% CI: 1-3.7). A similar association was found for both the CC genotype and C allele of the NLRP3 rs35829419 (OR: 2.78 and 2.73, respectively) and for the TT genotype and T allele of CARD8 rs2043211 (OR: 2.64 and 1.54, respectively). Yet, the IL-1*β* (rs1143629, rs1143634) and IL-18 (rs187238, rs1946518) polymorphisms did not show any significant association with response to interferon treatment. In conclusion, this study reports, for the first time, the association of genetic variations in NLRP3 with hepatitis C susceptibility and response to treatment in Egyptian patients. However, further large-scale studies are recommended to confirm our findings.

## 1. Introduction

Hepatitis C virus (HCV) is a global public health problem with more than 185 million infections worldwide [1]. The highest prevalence of HCV was reported in Egypt; approximately 12% of the Egyptian population are HCV positives, and 10% are chronically infected [2].

Hepatitis C virus often causes persistent infection in humans, which leads to chronic hepatitis in up to 60–80% of patients and can progress to liver fibrosis, cirrhosis, and eventually hepatocellular carcinoma [3].

A growing body of evidence has suggested that proinflammatory cytokines (IL-1*β* and IL-18) play a substantial role in both acute and chronic hepatitis C [4, 5]. This finding was supported by previous studies that reported elevated IL-1*β* (interleukin-1*β*) levels in HCV-related liver diseases than other forms of liver damage [6].

In acute infection, these cytokines have an antiviral effect [7–9] and persuade the expression of interferon-stimulated genes (ISGs) to increase the effectiveness of interferon actions [10–12].

However, IL-1*β* modulates various cellular processes in chronic HCV infection, including induction of COX-2, nitric oxide, and TNF-*α* [13]. The persistent production of IL-1*β* by hepatic macrophages in chronic HCV patients would then help to recruit immune cells to the liver causing inflammation which also affects the response to interferon therapy [14, 15].

Interleukin-1*β* is synthesized as pro-IL-1*β* that is converted into the active form by the inflammasome, which is a multiprotein complex consisting mainly of a Nod-like receptor (NLR), adaptor molecule, and procaspase-1. The recruitment of the inflammasome results in the activation of caspase-1, which cleaves pro-IL-1*β* and pro-IL-18 into their mature form [16].

Recent studies reported production of mature IL-1*β* through the NLRP3 inflammasome assembly in hepatic macrophages and hepatocytes infected with HCV [5, 17]. Furthermore, the activity of this inflammasome is regulated by the caspase recruitment domain-containing protein 8 (CARD8) [18].

The combination of pegylated interferon alpha (PEG-IFN) and ribavirin (RBV) is used for treatment of chronic HCV in Egypt. The main goal of HCV therapy is to produce a sustained virological response (SVR), which is defined as the absence of HCV RNA in the serum at the end of treatment and 6 months later [19]. Unfortunately, the rate of SVR is around 50% in HCV genotype 4-infected patients [20]. Of note, genotype 4 is the major HCV genotype (>90%) among the Egyptian patients [21, 22].

The host genetic variations play a pivotal role in susceptibility to HCV infection and fate of viral infection. Several polymorphisms within cytokine genes such as IL-18, IFN, IL-10, and IL-28 have been reported to be associated with HCV susceptibility and fate of the disease [23, 24]. Despite that NLRP3 gene polymorphisms have been found to be associated with susceptibility to HIV and human T-lymphotropic virus 1 infection [25, 26], their association with HCV infection is still ambiguous. So, the objective of the current study was to investigate the association of nine single-nucleotide polymorphisms in four inflammasome-related genes (NLRP3, IL-1*β*, IL-18, and CARD8) with susceptibility to HCV infection and outcome.

## 2. Material and Methods

### 2.1. Participants

This study included 201 chronic HCV patients recruited from the National Hepatology and Tropical Medicine Research Institute (NHTMRI), Cairo, Egypt. In addition, 95 healthy subjects were recruited as sex- and age-matched controls. The study was carried out from 2014 to 2016.

The study had been approved by the Research Ethical Committee (REC) of National Hepatology and Tropical Medicine Research Institute, serial number 11-2014, date 13 July 2014. This committee works according to the Declaration of Helsinki for human subjects (2008). Moreover, an informed consent was obtained from all the participants. The study has been conducted in accordance with national protocols for the treatment of chronic hepatitis C, which has been issued by the National Committee for Control of Viral Hepatitis, Ministry of Health in Egypt.

The selection criteria for chronic HCV patients were the positive HCV Ab and positive HCV viremia at the time of diagnosis. Liver biopsy was conducted for every patient to assess the degree of fibrosis and for exclusion of other concurrent liver diseases. The exclusion criteria were other hepatic viral infections, schistosomiasis, autoimmune diseases, hematological disorders, alcoholic liver disease, obesity-induced liver disease, renal disease, poorly controlled diabetes, and immunologically mediated disease (ulcerative colitis, Crohn's disease, systemic lupus erythematous, scleroderma, severe psoriasis, and rheumatic arthritis). Additionally, alcohol > 80 g/day and intravenous or inhaled drug abuse were also excluded. For healthy controls, all were negative for HCV Ab, HIV, HBV, and no other concurrent diseases.

Chronic hepatitis C patients received 48 weeks of treatment with combination therapy, according to the protocol used in NHTMRI, which is composed of IFN administrated subcutaneously in a dose of 1.5 *μ*g/kg once per week (dosage modified in case of cytopenia or symptomatic adverse events during treatment) and 1000–1200 mg of oral ribavirin per day. After discontinuation of treatment, they were followed for another 24 weeks. To determine the response to combination therapy, sera were tested for HCV RNA at the end of therapy and after 6 months of therapy. The chronic HCV patients were classified into two groups: the responder group included 86 subjects which achieved SVR defined as clearance of HCV RNA from serum at the end of treatment and for 24 weeks after the end of treatment.

The second group consisted of 115 “nonresponders/relapsers.” The nonresponder could not achieve end-of-treatment virological response (ETVR). Relapsers could successfully achieve ETVR but fail to achieve SVR (reappearance of HCV RNA).

A complete medical history and biochemical parameters were obtained for all the patients (height and weight to estimate BMI, age, ALT, AST, AFP, total bilirubin, ALP, albumin, Hb, TLC, the degree of liver fibrosis, platelet count, and creatinine); all parameters were initial before the start of treatment. Additionally, the viral load at baseline, week 12, week 24, week 48 (the end of the treatment), and 6 months after the end of treatment were also obtained.

### 2.2. Blood Sample and DNA Isolation

A venous blood sample of 2 mL was drawn from every participant. The blood sample was collected in sterile anticoagulant tubes. Genomic DNA was extracted from EDTA whole blood using PureLink® Genomic DNA Mini Kit (Invitrogen, USA).

### 2.3. Genetic Polymorphism Detection

Nine SNPs, three within the NLRP3 gene (rs1539019, rs35829419, and rs10754558), two within IL-1*β* (rs1143629, rs1143634), two within IL-18 (rs187238, rs1946518), and two within the CARD8 gene (rs6509365, rs2043211), were genotyped in this study. The genotyping was performed using TaqMan® Predesigned SNP Genotyping Assay (40x) (Catalog Number: 4351379); Invitrogen® by life technologies®; Applied Biosystems; USA. For the master mix, we used TaqMan® Universal Master Mix II, no UNG (2x) (Catalog Number: 4440043); Invitrogen® by life technologies®; Applied Biosystem; USA.

The PCR for detection of SNP was carried out according to the manufacturer's instructions using Rotor-Gene®, Qiagen, Real-Time PCR, USA. Allelic discrimination was performed using Rotor–Gene Q Software version: 2.0.2 (Build 4).

### 2.4. Statistical Analysis

The sample size was calculated using OpenEpi info (http://www.openepi.com) with the following parameters: confidence interval (CI) = 95%, case : control proportion = 1 : 1, statistical power *β* = 0.80, and minor allele frequency (MAF) estimated using Haploview bioinformatics software version 4.2, OR > 2. Relative excess heterozygosity (REH) was calculated to inspect the harmony of genotype frequencies within the control groups with the Hardy-Weinberg equilibrium (HWE) directly according to Wellek et al. [27]. In addition, *p* values from the standard exact HWE lack-of-fit test using 100,000 permutations (*p* = 0.05) were also studied. HWE analysis was performed using R 3.4.0 [28] with the “Hardy Weinberg version 1.5.8” package [29].

Risk assessment statistical analysis was conducted by SPSS version 21 (SPSS, Chicago, IL, USA). Continuous variables of demographic and anthropometric characteristics were compared using Student's *t*-test. The genotype and allele frequencies were calculated using the gene counting method. Univariate comparisons of categorical variables were performed with the chi–square test. Allele frequencies and genotype distributions between the studied groups were compared by the chi-square test and Fisher's exact test when expected value < 5 were found for any cell. Unadjusted (crude or exposure) odds ratio and 95% confidence interval (CI) were estimated. The nominal level of statistical significance for all analyses was *p* < 0.05.

The pairwise linkage disequilibrium between SNPs located on the same chromosomes (*D*′, *r*^2^) and haplotype proportions were estimated using Haploview bioinformatics software version 4.2. The correlation between the linkage disequilibrium haplotypes and HCV susceptibility were estimated using the SHEsis program (http://analysis.bio-x.cn/myAnalysis.php).

## 3. Results

Nine single-nucleotide polymorphisms in four inflammasome genes were genotyped in 201 chronic HCV patients and 95 healthy controls. The general characteristics of patients and controls are displayed in Table 1. The patients and the controls were age-, sex-, and BMI-matched.

The SNP characteristics are listed in Table 2. No significant deviation from the Hardy-Weinberg equilibrium was found in any of the studied SNPs in the healthy control group, indicating that the genotypes were appropriately assigned.

The genotypes and allele frequencies of all the studied SNPs are shown in Table 3. The HCV patients revealed a significantly higher AC genotype frequency for the NLRP3 rs1539019 compared to the healthy control group (OR 2.1, 95% CI: 1.3-3.5). On the other hand, the genotype CC was associated with significantly lower risk of chronic HCV infection (OR 0.33, 95% CI: 0.17-0.62).

Additionally, the frequencies of the CC genotype and C allele of the NLRP3 rs35829419 were significantly higher in the HCV patients compared to the healthy control group (OR 6.05, 95% CI: 3.4-10.66; OR 4.59, 95% CI: 2.79-7.55, respectively). Nevertheless, genotype CA and allele A were associated with decreased risk of HCV positivity. For NLRP3 rs10754558, no significant differences in genotypes or allele frequencies were found between the HCV patients and the healthy control group (Table 3).

For IL-1*β* rs1143629, the AA genotype was significantly more frequent in HCV patients (OR = 1.7). However, the IL-1*β* rs1143634 polymorphism revealed no association with HCV infection.

In contrast to the IL-18 rs187238, which did not show any association with HCV infection, genotype GG and allele G in the rs1946518 of the same gene were significantly more frequent in the healthy control indicating a protective effect against infection (OR: 0.55 and 0.61, respectively). However, the T allele of this SNP was associated with increased risk of HCV positivity (OR: 1.63).

On the other hand, the SNPs in the adaptor molecule CARD8 (rs6509365 and rs2043211) did not reveal any significant association with HCV infection (Table 3).

**Figure 1:**
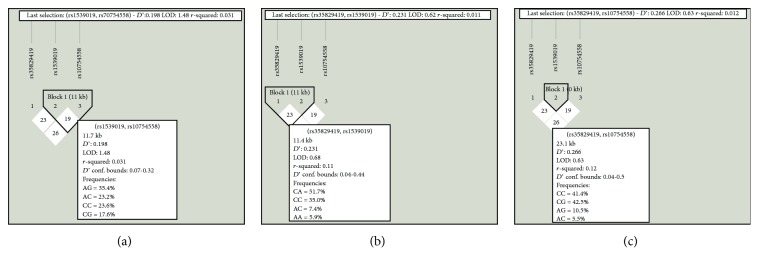
Pairwise linkage disequilibrium analysis of the studied SNPs located within NLRP3. (a) rs1539019 and rs10754558 are inherited independently (*D*′ = 0.19 and *r*^2^ = 0.31). (b) rs35829419 and rs1539019 are in linkage disequilibrium (*D*′ = 0.23 and *r*^2^ = 0.01). (c) rs35829419 and rs10754558 are inherited separately (*D*′ = 0.26 and *r*^2^ = 0.01).

**Figure 2:**
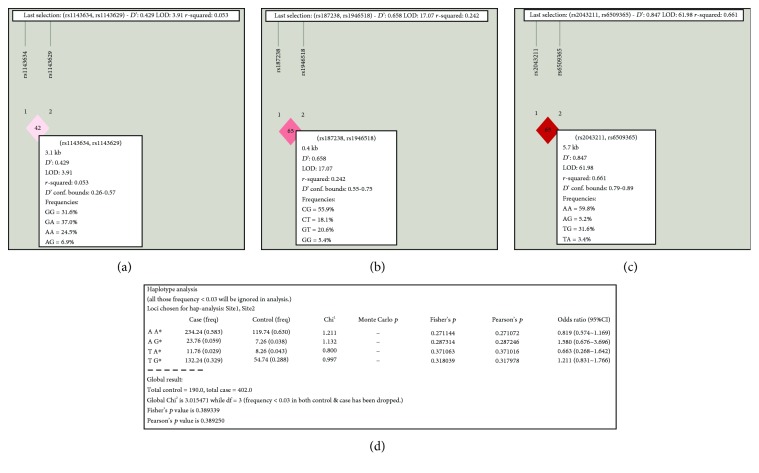
Pairwise linkage disequilibrium analysis of the studied SNPs located within IL-1*β*, IL-18, and CARD8, respectively. (a) rs1143629 and rs1143634 within IL-1*β* are inherited independently (*D*′ = 0.43 and *r*^2^ = 0.05). (b) rs187238 and rs1946518 within IL-18 are in linkage disequilibrium (*D*′ = 0.67 and *r*^2^ = 0.24). (c) rs2043211 and rs6509365 are in linkage equilibrium (*D*′ = 0.85 and *r*^2^ = 0.66). (d) The correlation of haplotypes of the studied SNPs within CARD8 with HCV susceptibility using the SHEsis program: no haplotype is found to play a role to HCV susceptibility. df = degree of freedom; CI = confident interval. *p* < 0.01 is considered significant.

The pairwise linkage disequilibrium analysis for the studied SNPs within NLRP3 is shown in Figure 1, and those within IL-1*β*, IL-18, and CARD8 are presented in Figure 2. It has been disclosed that the studied SNPs within NLRP3, IL-1*β*, and IL-18 were in linkage equilibrium. Furthermore, rs6509365 and rs2043211 within CARD8 were found to be in linkage disequilibrium (*D*′ = 0.85 and *r*^2^ = 0.661). This means that the latter SNPs were inherited as haplotypes. Further investigation reveals that none of these haplotypes associated with HCV susceptibility as shown in Figure 2(d).

As the patients recruited for this study already finished their interferon therapy protocols, we decided to test the association between the polymorphisms in the selected SNPs and the response to the interferon treatment. Hereafter, the HCV patients were classified based on their responses to interferon therapy into nonresponders (NR) and sustained virological responders (SVR) as shown in Table 4.

The patients with SVR showed a significantly lower AA genotype frequency of the NLRP3 rs1539019 compared to the nonresponder group (OR: 1.95). However, the frequencies of the CC genotype and C allele of the NLRP3 rs35829419 were significantly higher in the nonresponder group compared with the SVR group suggesting their association with the increased risk of lack of response to interferon treatment (OR: 2.78 and 2.73, respectively). On the other hand, the CA genotype and the A allele were associated with sustained response to IFN therapy (OR: 0.39, 0.37, respectively). No significant differences were found in the genotypes or alleles frequencies in the NLRP3 rs10754558 between the SVR and NR groups. For the IL-1*β* (rs1143629, rs1143634) and IL-18 (rs187238, rs1946518) polymorphisms, no significant association was found in terms of the response to IFN treatment.

When considering the CARD8 polymorphisms, the frequencies of the TT genotype and T allele of CARD8 rs2043211 were significantly higher in the NR group (OR: 2.64 and 1.54, respectively) making them associated with risk of failure of IFN treatment. However, the A allele of the same SNP was associated with more response to IFN treatment (OR: 0.65).

Upon classification of HCV patients based on the degree of liver fibrosis, we cannot find any significant difference in either genotypes or alleles frequencies of any of the studied SNPs (Table 5). This indicates lack of association between the studied inflammasome genes' polymorphisms and the severity of fibrosis.

## 4. Discussion

The inflammation is known to be a major player in the progression of the HCV-associated liver diseases. Recently, many studies have shown that the NLRP3 inflammasome is implicated in recognizing HCV and eliciting the subsequent innate immune and inflammatory responses [5, 17, 30]. The genetic factors might affect the susceptibility, severity, and chronicity of HCV infection [31]. To the best of our knowledge, this is the first study investigating the association of polymorphisms in NLRP3 and CARD8 genes with the risk of HCV infection and its response to IFN therapy. The targeted SNPs in our study were chosen based on the previous publications [25, 26].

We studied three SNPs in the NLRP3 gene, and we found a statistically significant association between rs1539019 and rs35829419 polymorphisms and HCV infection. Our data demonstrated that the NLRP3 rs35829419 C>A polymorphism was associated with increased risk to HCV infection. Mounting efforts have been devoted for investigating the effect of the genetic variation in NLRP3 on the susceptibility to various diseases. Several studies demonstrated the association of NLRP3 rs35829419 C>A polymorphism with the increased risk of colorectal cancer, HIV-1 infection, rheumatoid arthritis, leprosy, and atopic dermatitis [32–36].

Of note, a recent study found no correlation between the rs35829419 in NLRP3 and the renal damage caused by HCV-related cryoglobulinemia [37]. However, all the subjects recruited in this study were HCV patients (with or without renal damage) and no healthy control group was included. Thus, they did not test the association between the rs35829419 polymorphism and chronic HCV infection.

Interestingly, in our results the A allele of rs35829419 was more prevalent in the healthy control and the SVR groups indicating a protective effect of this allele against HCV infection. Recently, studies revealed that the NLRP3 Q705K (rs35829419) polymorphism is a gain-of-function change which results in more favourable innate immune response and this may provide a partial explanation for the protective effect of the A allele [38].

The association between the NLRP3 rs1539019 polymorphism and HCV infection is in the same line with a study demonstrating a strong association between the NLRP3 rs1539019 polymorphism and pneumoconiosis in Chinese coal workers [39]. Additionally, Dehghan et al. reported that rs1539019 is associated with the circulation fibrinogen levels and consequently the cardiovascular disease risk [40]. On the other hand, a study on a Japanese population reported no statistically significant association between the rs1539019 polymorphism and the risk of essential hypertension [41]. Notably, the NLRP3 rs1539019 is an intronic polymorphism whose function is not fully understood. However, several studies have reported that many transcription factors bind to intronic sites that may play a role in regulating the gene expression.

Interleukin-1 beta is a proinflammatory cytokine which is secreted by monocytes and macrophages and play a pivotal role in the innate immune response to infection [42]. There is mounting evidence that HCV patients have higher plasma IL-1*β* levels compared to healthy subjects emphasizing the implication of IL-1*β* in HCV pathogenesis [17]. However, the expression of IL-1*β* (mRNA levels) in the liver biopsies of chronic hepatitis C patients was found to be comparable to the controls [43]. In our study, the IL-1*β* rs1143629 revealed a significant association with HCV infection. In agreement with our findings, rs1143629 has been reported to be associated with malaria [44] and HIV-1 susceptibility [33]. Being an intronic SNP whose function is not fully understood does not exclude the importance of rs1143629 in influencing the IL-1*β* gene expression.

On the other hand, the IL-1*β* rs1143634, which was shown recently to be associated with advanced fibrosis in nonalcoholic steatohepatitis in Caucasians, did not reveal any association with the fibrosis degree in HCV patients in our study. rs1143634 is a synonymous SNP (F105F) in exon 5 of IL-1*β* whose function is not completely elucidated; yet, it was found to be associated with several diseases including HIV-1 [33] and periodontitis [45, 46]. However, no significant difference in rs1143634 genotyping or allele frequencies was detected in our study between HCV patients and healthy controls.

Interestingly, our results revealed a significant association between IL-18 rs1946518 and the susceptibility to HCV infection, but no significant association was observed for the IL-18 rs187238 polymorphism. Notably, both SNPs reside in the promoter region of the IL-18 gene and affect its expression and activity by disruption of transcription factor-binding sites [47, 48].

The proinflammatory cytokine IL-18 was reported to be involved in the HCV pathogenesis [49, 50]. In many studies, the IL-18 serum levels of HCV patients were significantly correlated with degree of liver injury and response to antiviral treatment [51, 52].

Our results showed that the T allele of IL-18 rs1946518 was associated with susceptibility to HCV; however, it was not associated with either response to treatment or fibrosis degree. Despite that there are many published studies which investigated the effect of IL-18 polymorphisms and the HCV outcome, the published data are very contradictory. A study conducted on Tunisian HCV patients reported a significant association between IL-18 polymorphism (rs1946518) and the severity of HCV [53]. A similar finding was reported in a study conducted on Indian patients [54]. However, a recent study stated that IL-18 rs1946518 polymorphism could not predict the outcome of the response of a combined interferon/ribavirin treatment in Egyptian HCV patients [55], which comes with agreement with our findings. The different findings among the studies could be explained by the differences in HCV genotypes and the ethnic groups.

The adaptor protein CARD8 is a crucial regulator of IL-1*β* production via inhibition of caspase-1 and/or inhibition of NF*κ*B-mediated gene expression [56, 57]. To the best of our knowledge, our study is the first study investigating the effect of genetic variations in CARD8 on HCV pathogenesis and progression. Our results revealed no association between the rs6509365 or rs2043211 polymorphism with the susceptibility to HCV or the severity of the disease (fibrosis degree). However, only CARD8 rs2043211 was associated with the response to interferon therapy in our study. Previous studies have reported that CARD8 rs2043211 was found to be associated with susceptibility to gout [58], nodular melanoma [59], and decreased risk of ankylosing spondylitis [60]. Nonetheless, CARD8 rs2043211 could not predict the occurrence or progression of coronary artery diseases [61] or the susceptibility and outcome of meningitis [62]. The CARD8 rs2043211 (C10X) polymorphism leads to a nonfunctional truncated CARD8 and subsequently more active inflammasome and prolonged inflammation [38, 63].

We are aware that our study has some limitations including the small number of patients enrolled from only one population (Egyptian) which makes it hard to generalize the conclusions of our study. So, a consortium multicenters' study with patients from different countries all over the world should be considered in order to draw a general conclusion on the correlation of the genetic variations in inflammasome genes and susceptibility to HCV. Also, this study did not include the spontaneously resolved patients (anti-HCV positive, negative serum viremia), which is another limitation. However, most of Egyptian patients with spontaneous clearance of HCV skip the proper diagnosis, and this made the recruitment of this group almost impossible. Moreover, we are also aware that some of the HCV treatment protocols have been shifted to the directly acting antiviral agents (DAA), which achieve a better sustained virologic response. Unfortunately, when we conducted our study, the DAA treatment protocol was just starting in Egypt with a very limited number of patients which makes it very hard to recruit them in our study. However, in the future we are planning to investigate the genetic variation in NLRP3 in correlation with the DAA response in HCV patients.

In conclusion, the present study reported, for the first time, that the genetic polymorphisms in NLRP3 (rs1539019 and rs35829419) are associated with hepatitis C susceptibility and response to treatment in Egyptian patients. Thus, our work highlights the importance of the genetic variations of innate immune factors for the predisposition to HCV infection and outcome. However, further large-scale studies are recommended to confirm our findings.

## Figures and Tables

**Table 1:** General characteristics of the studied groups.

	C *N* = 95	P *N* = 201	SVR *N* = 86	NR *N* = 115
Male/female	56/39	117/84	59/27^∗^	58/57^∗^
Age (mean ± SE) years	37 ± 1.2	39.8 ± 0.85	38.4 ± 1.35	41 ± 1
BMI	26 ± 0.24	27 ± 0.42	27 ± 0.5	26.8 ± 0.6
ALT level (U/L, mean ± SE)		54 ± 2.5	54 ± 3.8	53 ± 3.3
Basal HCV RNA level (log copies/mL, mean ± SE)		5.5 ± 0.1	5.4 ± 0.1	5.6 ± 0.1
Fibrosis				
F1		107 (57.2%)	57 (67.9%)	50 (48.5%)
F2		38 (20.3%)	15 (17.9%)	23 (22.3%)
F3		42 (22.5%)	12 (14.3%)	30 (29.1%)

C = healthy control; P = HCV patients; BMI = body mass index; ALT = alanine transaminase; SVR = sustained virological response; NR = nonresponder; F = fibrosis. Quantitative data are represented as mean ± SE, qualitative data are represented as number and frequency, ^∗^*p* < 0.01 is considered significant.

**Table 2:** SNP characteristics and population.

Gene	SNP ID	Alleles (major/minor)	Ch no/position	mRNA position	Protein position	Change in amino acid	Relative excess heterozygosity (95% confidence interval) ^a^	*p* value HWE^b^
NLRP3	rs1539019	A/C	1: 247436999	—	—	Intron	0.7 (0.47-1.06)	0.118
	rs35829419	C/A	1: 247425556	2859	705	Q705K	1.8 (0.95-3.53)	0.103
	rs10754558	G/C	1: 247448734	3745	—	3′UTR	0.8 (0.49-1.19)	0.298

IL-1B	rs1143629	A/G	2: 112835941	—	—	Intron	1.2 (0.77-1.75)	0.654
	rs1143634	G/A	2: 112832813	402	105	F105F	0.6 (0.4-0.97)	0.055

IL-18	rs187238	C/G	11: 112164265	—	—	Intron	0.9 (0.57-1.57)	0.976
	rs1946518	G/T	11: 112164735	—	—	Intron	0.8 (0.51-1.29)	0.483

CARD8	rs6509365	A/G	19: 48240212	—	—	Intron	0.9 (0.58-1.43)	0.81
	rs2043211	A/T	19: 48234449	541	102	C10X	0.6 (0.4-0.97)	0.055

^a^2-sided *p*-value. *p* < 0.05 is considered significant. ^b^*p*-value of the exact Hardy–Weinberg equilibrium lack-of-fit test. Q: glutamine; K: lysine; F: phenylalanine; C: cysteine; X: termination.

**Table 3:** Relation of Allele and genotype frequencies of SNPs with the HCV susceptibility.

Gene	SNP ID		P *N* (%)	C *N* (%)	OR	95% CI	*p* value
NLRP3	rs1539019	AA	60 (29.9%)	31 (32.6%)	0.88	0.52-1.5	0.63
		AC	120 (59.7%)	39 (41.1%)	2.13	1.3-3.5	*0.003*
		CC	21 (10.4%)	25 (26.3%)	0.33	0.17-0.62	*0.0006*
		A	104 (59.7%)	51 (53.2%)	1	0.64-1.68	0.87
		C	96 (40.3%)	49 (46.8%)	0.96	0.59-1.55	0.87
	rs35829419	CC	173 (86.1%)	48 (50.5%)	6.05	3.4-10.66	*<0.0001*
		CA	27 (13.4%)	44 (46.3%)	0.18	0.1-0.32	*<0.0001*
		AA	1 (0.5%)	3 (3.2%)	0.15	0.016-1.5	0.11
		C	373 (92.8%)	140 (73.7%)	4.59	2.79-7.55	*<0.0001*
		A	29 (7.2%)	50 (26.3%)	0.22	0.13-0.36	*<0.0001*
	rs10754558	GG	39 (26.5%)	25 (31.7%)	0.78	0.43-1.42	0.42
		GC	78 (53.1%)	34 (43%)	1.5	0.86-2.6	0.15
		CC	30 (20.4%)	20 (25.3%)	0.76	0.4-1.44	0.4
		G	156 (53.1%)	84 (53.2%)	1	0.68-1.47	0.98
		C	138 (46.9%)	74 (46.8%)	1	0.68-1.48	0.98

IL-1*β*	rs1143629	AA	86 (42.8%)	29 (30.5%)	1.7	1-2.86	*0.04*
		AG	84 (41.8%)	50 (52.6%)	0.65	0.4-1.1	0.08
		GG	31 (15.4%)	16 (16.8%)	0.9	0.47-1.74	0.76
		A	256 (63.7%)	108 (56.8%)	1.33	0.94-1.9	0.11
		G	146 (36.3%)	82 (43.2%)	0.75	0.53-1.1	0.11
	rs1143634	GG	97 (48.3%)	47 (49.5%)	0.95	0.58-1.55	0.85
		GA	85 (42.3%)	33 (34.7%)	1.38	0.83-2.3	0.22
		AA	19 (9.5%)	15 (15.8%)	0.56	0.27-1.15	0.11
		G	279 (69.4%)	127 (66.8%)	1.13	0.78-1.63	0.53
		A	123 (30.6%)	63 (33.2%)	0.89	0.61-1.29	0.53

IL-18	rs187238	CC	102 (50.7%)	52 (54.7%)	0.85	0.52-1.39	0.52
		CG	94 (46.8%)	36 (37.9%)	1.44	0.87-2.37	0.15
		GG	5 (2.5%)	7 (7.4%)	0.32	0.1-1.04	0.058
		C	298 (74.1%)	140 (73.7%)	1.02	0.69-1.52	0.91
		G	104 (25.9%)	50 (26.3%)	0.98	0.66-1.45	0.91
	rs1946518	GG	70 (34.8%)	47 (49.5%)	0.55	0.33-0.9	*0.017*
		GT	92 (45.8%)	37 (38.9%)	1.32	0.8-2.17	0.27
		TT	39 (19.4%)	11 (11.6%)	1.84	0.9-3.77	0.097
		G	232 (57.7%)	131 (69%)	0.61	0.43-0.89	*0.009*
		T	170 (42.3%)	59 (31%)	1.63	1.13-2.34	*0.009*

CARD8	rs6509365	AA	74 (36.8%)	44 (46.3%)	0.68	0.41-1.1	0.12
		AG	98 (48.8%)	40 (42.1%)	1.31	0.8-2.14	0.28
		GG	29 (14.4%)	11 (11.6%)	1.29	0.61-2.7	0.5
		A	246 (61.2%)	128 (67.4%)	0.76	0.53-1.1	0.15
		G	156 (38.8%)	62 (32.6%)	1.31	0.91-1.8	0.15
	rs2043211	AA	82 (40.8%)	47 (49.5%)	0.7	0.43-1.15	0.16
		AT	94 (46.8%)	33 (34.7%)	1.65	1-2.73	0.05
		TT	25 (12.4%)	15 (15.8%)	0.76	0.38-1.51	0.43
		A	258 (64.2%)	127 (66.8%)	0.89	0.62-1.28	0.53
		T	144 (35.8%)	63 (33.2%)	1.13	0.78-1.62	0.53

IFN = interferon; OR = odds ratio; CI = confidence interval; C = healthy control, P = HCV patients, SVR = sustained virological response, NR = nonresponder. All SNPs were characterized for 201 HCV patients (except rs10754558 is performed only on 147 patients) and 95 controls. Significant *p* values are in italics. *p* < 0.05 is considered significant.

**Table 4:** Relation of allele and genotype frequencies of SNPs with the IFN response.

Gene	SNP ID		SVR *N* (%)	NR *N* (%)	OR	95% CI	*p* value
NLRP3	rs1539019	AA	19 (22.1%)	41 (35.7%)	1.95	1-3.7	*0.04*
		AC	56 (65.1%)	64 (55.7%)	0.67	0.38-1.2	0.18
		CC	11 (12.8%)	10 (8.7%)	0.65	0.26-1.6	0.35
		A	94 (54.7%)	146 (63.5%)	1.44	0.96-2.16	0.07
		C	78 (45.4%)	84 (36.5%)	0.69	0.46-1.04	0.07
	rs35829419	CC	68 (79.1%)	105 (91.3%)	2.78	1.2-6.4	*0.016*
		CA	17 (19.8%)	10 (8.7%)	0.39	0.17-0.9	*0.026*
		AA	1 (1.2%)	0	0.25	0.01-6.1	0.39
		C	153 (89%)	220 (95.7%)	2.73	1.24-6	*0.013*
		A	19 (11%)	10 (4.3%)	0.37	0.17-0.81	*0.013*
	rs10754558	GG	19 (26.4%)	20 (26.7%)	1.01	0.49-2.1	0.97
		GC	38 (52.8%)	40 (53.3%)	1.02	0.53-1.9	0.95
		CC	15 (20.8%)	15 (20%)	0.95	0.43-2.1	0.9
		C	68 (47.2%)	70 (46.7%)	0.98	0.62-1.5	0.92
		G	76 (52.8%)	80 (53.3%)	1.02	0.65-1.6	0.92

IL-1*β*	rs1143629	AA	36 (41.9%)	50 (43.5%)	1.07	0.61-1.88	0.82
		AG	37 (43.0%)	47 (40.9%)	0.92	0.52-1.61	0.76
		GG	13 (15.1%)	18 (15.7%)	1.04	0.48-2.26	0.92
		A	109 (63.4%)	147 (63.9%)	1.02	0.68-1.54	0.91
		G	63 (36.6%)	83 (36.1%)	0.98	0.65-1.47	0.91
	rs1143634	GG	44 (51.2%)	53 (46.1%)	0.82	0.47-1.43	0.48
		GA	36 (41.9%)	49 (42.6%)	1.03	0.59-1.82	0.92
		AA	6 (7.0%)	13 (11.3%)	1.7	0.62-4.67	0.3
		G	124 (72.1%)	155 (67.4%)	0.8	0.52-1.23	0.31
		A	48 (27.9%)	75 (32.6%)	1.25	0.81-1.93	0.31

IL-18	rs187238	CC	48 (55.8%)	54 (47.0%)	0.7	0.4-1.23	0.21
		CG	37 (43.0%)	57 (49.6%)	1.3	0.74-2.28	0.36
		GG	1 (1.2%)	4 (3.5%)	3.06	0.33-28	0.32
		C	133 (77.3%)	165 (71.7%)	0.74	0.47-1.18	0.21
		G	39 (22.7%)	65 (28.3%)	1.34	0.85-2.12	0.21
	rs1946518	GG	30 (34.9%)	40 (34.8%)	1	0.55-1.79	0.99
		GT	41 (47.7%)	51 (44.3%)	0.87	0.5-1.53	0.64
		TT	15 (17.4%)	24 (20.9%)	1.25	0.61-2.55	0.54
		G	101 (58.7%)	131 (57%)	0.93	0.62-1.39	0.72
		T	71 (41.3%)	99 (43%)	0.93	0.62-1.39	0.72

CARD8	rs6509365	GG	12 (14.0%)	17 (14.8%)	1.07	0.48-2.38	0.89
		AG	38 (44.2%)	60 (52.2%)	1.38	0.79-2.42	0.26
		AA	36 (41.9%)	38 (33.0%)	0.69	0.38-1.22	0.2
		A	110 (64%)	136 (59.1%)	0.82	0.54-1.23	0.33
		G	62 (36%)	94 (40.9%)	1.23	0.82-1.84	0.33
	rs2043211	AA	40 (46.5%)	42 (36.5%)	0.66	0.37-1.17	0.15
		AT	40 (46.5%)	54 (47.0%)	1.02	0.58-1.78	0.95
		TT	6 (7.0%)	19 (16.5%)	2.64	1-6.92	*0.048*
		A	120 (69.8%)	138 (60%)	0.65	0.43-0.99	*0.04*
		T	52 (30.2%)	92 (40%)	1.54	1.01-2.34	*0.04*

IFN = interferon; OR = odds ratio; CI = confidence interval; SVR = sustained virological response; NR = nonresponder. All SNPs were characterized for 201 HCV patients (except rs10754558 is performed only on 147 patients) and 95 controls. Significant *p* values are in italics. *p* < 0.05 is considered significant.

**Table 5:** Influence of SNPs on the degree of liver fibrosis in HCV patients.

Gene	SNP ID	Genotype	F1 (107)	F2 (39)	F3 (42)	*p* value
NLRP3	rs1539019	AA	28 (26.2%)	17 (43.6%)	11 (29.4%)	0.95
		AC	64 (59.8%)	21 (53.8%)	28 (60.4%)	
		CC	15 (14%)	1 (2.6%)	3 (10.2%)	
		A	120 (56.1%)	55 (70.5%)	50 (59.5%)	0.08
		C	94 (43.9%)	23 (29.5%)	34 (40.5%)	
	rs35829419	CC	93 (86.9%)	34 (87.2%)	34 (81%)	0.74
		CA	13 (12.1%)	5 (12.8%)	8 (19%)	
		AA	1 (0.9%)	0 (0%)	0 (0%)	
		C	199 (93%)	91 (94.8%)	76 (90.5%)	0.7
		A	15 (7%)	5 (5.2%)	8 (9.5)	
	rs10754558	GG	25 (28.1%)	9 (30%)	5 (17.9%)	0.7
		GC	47 (52.8%)	16 (53.3%)	15 (53.5%)	
		CC	17 (19.1%)	5 (16.7%)	8 (28.6%)	
		G	97 (54.5%)	34 (56.7%)	25 (44.6%)	0.36
		C	81 (45.5%)	26 (43.3%)	31 (55.4%)	

IL-1*β*	rs1143629	AA	40 (37.4%)	23 (59%)	18 (42.9%)	0.21
		AG	49 (45.8%)	13 (33.3%)	18 (42.9%)	
		GG	18 (16.8%)	3 (7.7%)	6 (14.3%)	
		A	129 (60.3%)	59 (75.6%)	54 (64.3%)	0.52
		G	85 (39.7%)	19 (24.4%)	30 (35.7%)	
	rs1143634	GG	55 (51.4%)	14 (35.9%)	21 (50.0%)	0.4
		GA	43 (40.2%)	22 (56.4%)	16 (38.1%)	
		AA	9 (8.4%)	3 (7.7%)	5 (11.9%)	
		G	153 (71.5%)	50 (64.1%)	58 (69.1%)	0.48
		A	61 (28.5%)	28 (35.9%)	26 (30.9%)	

IL-18	rs187238	GG	1 (0.9%)	3 (7.7%)	1 (2.4%)	0.15
		CG	47 (43.9%)	17 (43.6%)	23 (54.8%)	
		CC	59 (55.1%)	19 (48.7%)	18 (42.9%)	
		C	165 (77.1%)	55 (70.5%)	59 (70.2%)	0.34
		G	49 (22.9%)	23 (29.5%)	25 (29.8%)	
	rs1946518	GG	40 (37.4%)	13 (33.3%)	11 (26.2%)	0.35
		GT	47 (43.9%)	16 (41.0%)	25 (59.5%)	
		TT	20 (18.7%)	10 (25.6%)	6 (14.3%)	
		G	127 (59.4%)	42 (53.9%)	47 (55.9%)	0.67
		T	87 (40.7%)	36 (46.1%)	37 (44.1%)	

CARD8	rs6509365	GG	11 (10.3%)	7 (17.9%)	8 (19.0%)	0.5
		AG	52 (48.6%)	20 (51.3%)	20 (47.6%)	
		AA	44 (41.1%)	12 (30.8%)	14 (33.3%)	
		A	140 (65.4%)	44 (56.4%)	48 (57.1%)	0.23
		G	74 (34.6%)	34 (43.6%)	36 (42.9%)	
	rs2043211	AA	47 (43.9%)	14 (35.9%)	17 (40.5%)	0.69
		AT	50 (46.7%)	18 (46.2%)	20 (47.6%)	
		TT	10 (9.3%)	7 (17.9%)	5 (11.9%)	
		A	144 (67.3%)	46 (59%)	54 (64.3%)	0.42
		T	70 (32.7%)	32 (41%)	30 (35.7%)	

Fibrosis degree was assessed using METAVIR score. The statistical analyses were conducted using the chi-square test. *p* < 0.05 is considered significant.

## Data Availability

The data used to support the findings of this study are available from the corresponding author upon request.

## Abbreviations

NLRP3: NOD-like receptor, pyrin domain-containing 3
IL-1*β*: Interleukin-1*β*
ISGs: Interferon-stimulated genes
PEG-IFN: Pegylated interferon alpha
CARD8: Caspase recruitment domain-containing protein 8
SVR: Sustained virological response
HWE: Hardy-Weinberg equilibrium
SNP: Single-nucleotide polymorphism.
